# Re: Letter regarding Pragmatism and Professionalism

**DOI:** 10.1308/rcsann.2022.0114

**Published:** 2022-10-31

Dear Mr Roche,

Thank you for your letter regarding the two duplicated case reports identified in 2021 and my subsequent editorial.^1,2,3^ I would like to thank you for the time, knowledge and interest you have taken with this. Your thoughts and comments have educated me on the complexities of investigating such cases.

We understand there are differing opinions regarding the efficacy and usefulness of the Committee on Publication Ethics (COPE) guidelines. Notwithstanding this, COPE is considered the industry standard, and *Annals* aims to refer to COPE in matters of academic publishing governance. Thus, we subsequently stated an expression of concern which was highlighted to readers in my editorial. Rather than being both judge and jury, I feel detailing an expression of concern allows the reader, both now and in the future, to decide the merits of the manuscript(s).

As a result of this case, we now use iThenticate^4^ anti-plagiarism software for every paper, rather than spot-checking. While ethical issues like this are ongoing for all academic journals, rest assured the Annals team will endeavour to be transparent with readers, authors and institutions in any such matters.

Kind Regards,

Benedict Rogers
